# A decade of civilian vascular trauma in Kosovo

**DOI:** 10.1186/1749-7922-7-24

**Published:** 2012-07-20

**Authors:** Luan Jaha, Tatjana Andreevska, Hajriz Rudari, Bekim Ademi, Vlora Ismaili-Jaha

**Affiliations:** 1 Department of Vascular Surgery, University Clinical Center of Kosovo, Prishtina, Republic of Kosovo; 2 Department of Thoracovascular surgery, University Clinical Center of Macedonia, Skopje, Republic of Macedonia

**Keywords:** Arterial trauma, Outcome, Kosovo

## Abstract

**Purpose:**

We sought to analyze the results of arterial injury management in a busy metropolitan vascular unit and risk factors associated with mortality and morbidity.

**Patients and methods:**

We analyzed 120 patient with arterial injury treated between year 2000 and 2010 at the University Clinical Center of Kosovo. Seven of these years were prospective and three retrospective study.

**Results:**

The mechanism of arterial injury was stabbing 46.66%, gunshot wounds in 31.66%, blunt in 13.33%, and landmine in 8.33%. The most frequently injured vessel was the superficial femoral artery (25%), followed by the brachial artery (20.9%), crural arteries (13.1%), forearm arteries (14.3%), iliac arteries (7.5%), abdominal aorta (3.3%), common femoral artery (3.3%) and popliteal artery (3.3%). Associated injuries including bone, nerve and remote injury (affecting the head, chest, or abdomen) were present in 24.2% of patients. The decision to operate was made based on the presence of “hard signs” of vascular trauma. Arterial reconstruction was performed in 90.8% of patients, 5.8% of patients underwent primary amputation and 3.2% died on the operation table. Overall survival rate was 95.8%.

**Conclusion:**

Injuries to the arteries are associated with significant mortality and morbidity. Mechanism of injury (blunt, gunshot, landmine or stub), hemodynamic stability at the admission, localization of injury, time from injury to flow restitution, associated injuries to the structures in the region and remote organs are critical factors influencing outcome.

## Patients and methods

All patients that suffered an injury to their vessels treated at the Emergency Center of the University Clinical Center of Kosovo (UCCK) between January 2001 and December 2010 are included in the study. UCCK is a busy vascular unit serving around 2,5 million people. It is the only vascular center in the Republic of Kosovo.

All demographic data, data on the type of injury, localization of injury, time from injury to the definite repair, data on clinical presentation at admission and hemodynamic stability of the injured, those on associated injury and existing comorbidities, are collected in standardized form. At the same form, we collect data on the mode of diagnostic evaluation, employed treatment employed and outcome.

Time to revascularization is defined as the period from the approximate time of injury to the time at which the patency of the injured vessel is restored at surgery. Arterial reconstruction was considered successful when the pulse distal to the reconstruction was present or if the continuity of the vessel was documented by angiography. Limb salvage is defined as the presence of a viable limb at one month after injury, regardless of functional outcome.

Statistical analysis is performed employing *t*-test for independent samples, Breakdown one-way ANOVA for symmetric distribution and Mann- Whitney *U* test, *X*2-test and Kruskal-Wallis for values of asymmetric distribution.

## Results

### Demographic data

Our study involved 120 patients with arterial trauma. Half of patients were 20 to 39 year old (52.5%) with a peak in age between 20 to 25 year. Every fifth patient (20%) was between 10 and 19 year old and every twelfth (10%) between 40 and 50 year old. Patients of other age groups were injured infrequently – only 5 were younger than 10 (4.2%), 8 (6.7%) were between 50 and 59 year old and other 8 (6.7%) older than 60 year in age.

The mean age of the patients in the study was 31.2 years (SD ± 15.5 yrs), ranging between 1 and 85 years. Using Mann Whitney test, we found no significant importance between the mean age and the gender of the patients (U = 557.5, P = 0.947 or P > 0.05), **(**Table 1**)**.

**Table 1:** Age and gender of the patients in study

**Age group**	**Gender**		**Total**	
	**F**	**M**		
	**N**	**N**	**N**	**%**
<10	1	4	5	4.2
10-19	2	22	24	20.0
20-29	2	30	32	26.7
30-39	1	30	31	25.8
40-49	2	10	12	10.0
50-59	1	7	8	6.7
60+	1	7	8	6.7
**Total**	**10**	**110**	**120**	**100.0**

### Mode of injury

**Figure 1:**
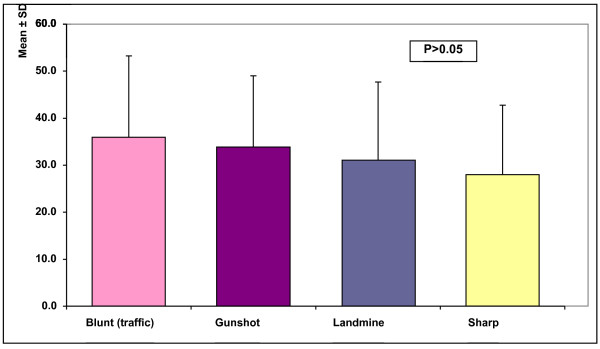
Age and mechanism of injury in patients in our study.

The mechanism of arterial injury was stabbing 46.66%, gunshot in 31.66%, blunt in 13.33%, and landmine in 8.33% (Figure 1).

The majority of the female patients in the study were in the group of patients that suffered blunt trauma (30% of all female patients in the study and 23.07% of all patients with blunt trauma). Female patients represented 5.55 of patients in the group that suffered gunshot injury and 9.43% of the patients that suffered sharp injury. None of the patients in the landmine group was female.

### Site of injury

The most frequently injured vessel was the superficial femoral artery (25%), followed by the brachial artery (20.9%), crural arteries (13.1%), forearm arteries (14.3%), iliac arteries (7.5%), abdominal aorta (3.3%), common femoral artery (3.3%) and popliteal artery (3.3%). Other arteries were injured less frequently.

**Figure 2:**
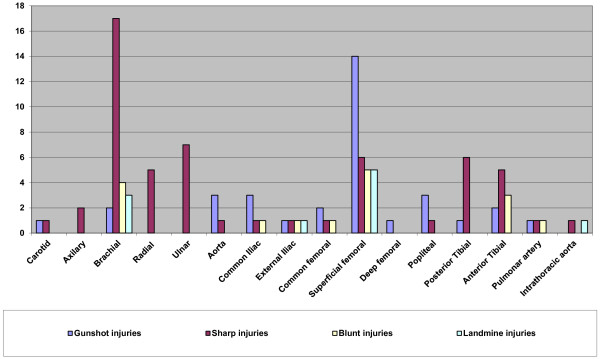
Anatomic distribution of injuries.

Evaluation of the data on the site of injury indicates that the superficial femoral artery was the most commonly injured in gunshot and injuries inflicted by landmines, while the brachial artery injuries inflicted by sharp objects. Superficial femoral artery and brachial artery were the equally frequent in blunt trauma (Figure 2).

### Associated injuries

Associated injuries including bone, nerve and remote injury (affecting the head, chest, or abdomen) were present in 24.2% of patients (Table 2). Such were all blunt and landmine injuries, 34.21% of the gunshot injuries and only 5.35% of the injuries inflicted by sharp objects. Evaluated statistically difference was significant (*X*^2^-test = 16.5, P = 0.001).

**Table 2:** Complexity and mechanism of injury

**Injury to the artery**	**Mode of injury**				**Total**	
	**Blunt**	**Gunshot**	**Landmine**	**Sharp**	**N**	**%**
Isolated	8	25	5	53	91	75.8
Complex	8	13	5	3	29	24.2
**Total**	**16**	**38**	**10**	**56**	**120**	**100.0**
*X*^2^-test	*X*^2^ = 16.5, P = 0.001					

### Clinical presentation and hemodynamic stability at the admission

Bleeding was the commonest clinical presentation in all four groups of injured (97/120 or 80.8%). Ischemia was less common (22/120 or 18.3%) and pulsatile hematoma was the least (1/120 or 0.8%). (Table 3).

**Table 3:** Clinical presentation of the injured at the admission

**Clinical Presentation**	**Mode of injury**				**Total**	
	**Blunt trauma**	**Gunshot injury**	**Landmine injury**	**Sharp object**	**N**	**%**
Bleeding	12	30	9	46	97	80.8
Hematoma	-	1	-	-	1	0.8
Ischemia	4	7	1	10	22	18.3
**Total**	**16**	**38**	**10**	**56**	**120**	**100.0**

The majority of the patients were admitted at the Emergency Center of the University Clinical Center hemodynamically stable (77/120 or 64.2%). Hemodynamically stable patients were especially in the group that suffered sharp vascular trauma (48/56 or 85.7%). Patients that suffered gunshot injury comprised the majority of the patient with hemodynamic instability at the admission (21/43 patients or 48.83% of all patients in shock). However, this was only a little more than half of all patients with gunshot injury (21/38 or 55.26%). In contrast 80% of patients that suffered landmine injury (8/10) where in the state of shock. In shock, at the admission, was almost every third patient that suffered blunt injury (6/16 or 37.5%) whiles the state of shock was less common for patients that suffered sharp vascular trauma (8/56 or 14.3%). Employing *X*2 test, we found high statistical correlation between hemodynamic stability and mode of injury (*X*^2^-test = 16.18, P = 0.001). (Table 4).

**Table 4:** Hemodynamic stability of the injured at the admission

**Hemodynamic stability**	**Mode of injury**				**Total**	
	**Blunt trauma**	**Gunshot injury**	**Landmine injury**	**Sharp injury**	**N**	**%**
Hypovolemic shock	6	21	8	8	43	35.8
Hemodynamic stability	10	17	2	48	77	64.2
**Total**	**16**	**38**	**10**	**56**	**120**	**100.0**
*X*^2^-test	*X*^2^ = 16.18, *P* = 0.001					

### The diagnostic workup

**Figure 3:**
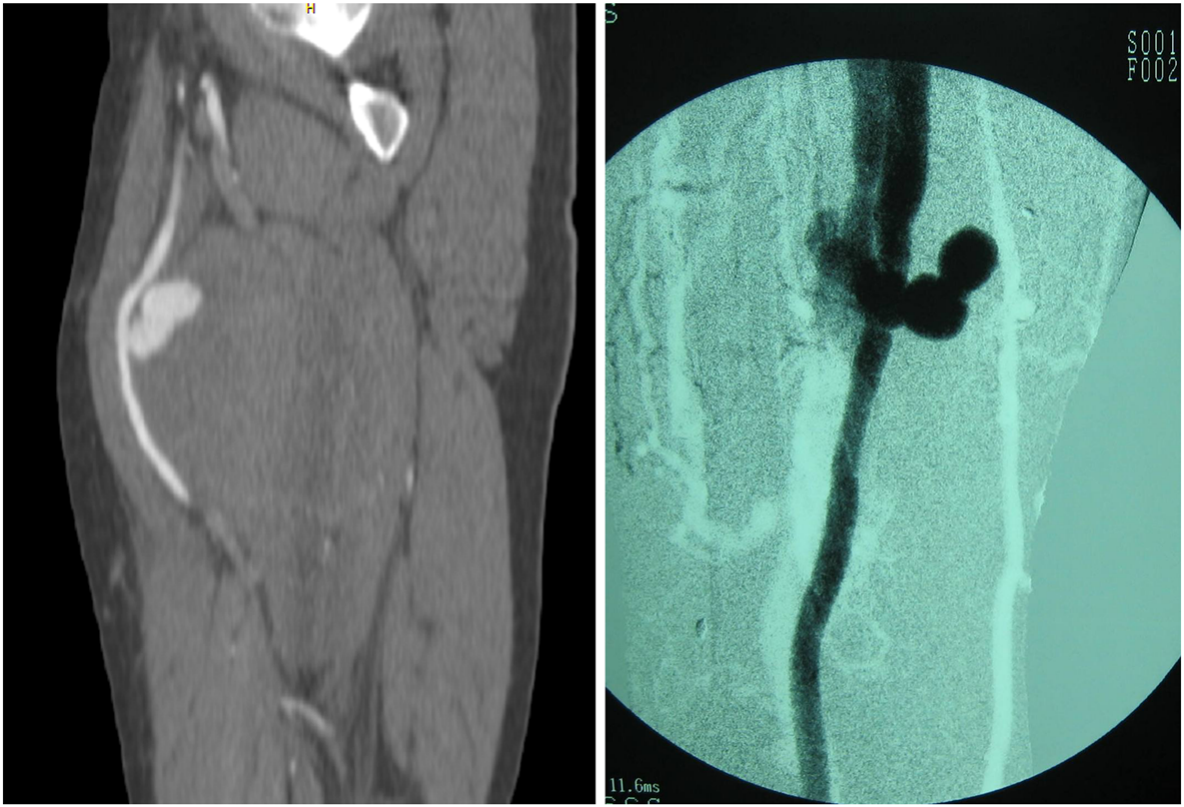
False aneurysm (a) and arteriovenous fistula (b) due to a non recognized arterial trauma.

In almost all patients, except in four (116/120 or 96.66%), the decision to operate was based on the presence of “hard signs” of vascular trauma. Although, performed only in about half of all trauma patients (63/120 or 52.5%), triplex scan was a powerful tool to support clinical decision. To confirm trauma to the vessels, we had to perform computerized angiotomography in four cases (4/120 or 3.33%). Two injuries were initially missed (2/120 or 1.66%) and presented later as false aneurysm and arteriovenous fistula (Figure 3).

### Mode of treatment

The vast majority of the patients underwent vascular reconstruction (109/120 or 90.8%). Seven of patients underwent primary amputation (7/120 or 5.8%), and four of the injured died at the operating theatre (4/120 or 3.3%). The majority of the death causalities belong to the group of patients that suffered gunshot injury (3/4 or 75% of death causalities, otherwise 3/38 or 7.9% of all patients that suffered gunshot injury) (Table 5).

**Table 5:** Patient according to the mode of treatment

**Type of reconstruction**	**Mode of injury**				**Total**	
	**Blunt trauma**	**Gunshot injury**	**Landmine injury**	**Sharp object**	**N**	**%**
Primary amputation	-	1	6	-	7	5.8
Death causalities	1	3	-	-	4	3.3
Vascular reconstruction	15	34	4	56	109	90.8
**Total**	**16**	**38**	**10**	**56**	**120**	**100.0**

### Surgical technique

End to end anastomosis was the most frequently employed surgical technique for treatment of our patients (70/120 or 58.3%), followed by autologous vein interposition (18/120 or 15.0%), lateral suture (12/120 or 10.0%), ligature of the injured artery (6/120 or 5.0%) and interposition of the synthetic graft (3/120 or 2.5%).

End to end anastomosis was the most commonly practiced in the group of patients that suffered gunshot injury and blunt injury (14/38 or 36.8% and 12/16 or 75.0%).

We employed vein interposition most commonly in patients with gunshot injury to their vessels – 10 of 18 vein interpositions belong to this group comprising every fourth patient in this group (26.4%).

Interposition of the synthetic graft was performed in only in 3 cases, all in the group of patients that suffered gunshot injury (3/120 or 2.5% of all patients in study or 3/38 or 7.9% of gunshot injured).

Half of lateral suture reconstruction were performed in the group of patients that suffered gunshot injury (6/12 or 50.0% of all patients with lateral suture, or 6/38 or 15.8% of the patients that suffered gunshot injury).

Ligature was practiced in six patients. In four cases - in patients with sharp vascular trauma (4/56 or 7.1% of the sharp vascular trauma), in one case - in patient with gunshot injury (1/38 or 2.6% of all gunshot injured) and in one - in blunt trauma (1/16 or 6.3% of all suffered blunt trauma) (Table 6).

**Table 6:** Patient in study according to the type of vascular reconstruction

**Type of reconstruction**	**Mode of injury**				**Total**	
	**Blunt injury**	**Gunshot injury**	**Landmine injury**	**Sharp vascular injury**	**N**	**%**
Synthetic graft	-	3	-	-	3	2.5
Autologous venous graft	1	10	2	5	18	15.0
Ligature	1	1	-	4	6	5.0
Lateral suture	1	6	1	4	12	10.0
End to end anastomosis	12	14	1	43	70	58.3
**Total**	**16**	**38**	**10**	**56**	**120**	**100.0**

## Discussion

In this study, we have reviewed our experience in dealing with civilian arterial trauma. In the light of standardizes management protocol, we sought to analyze factors influencing the outcome in patients. Although arterial trauma in this series was associated with low mortality rate and the high percentage of limb salvage, study indicates the importance of several factors for outcome.

*First,* it is the age of the patient. Our study indicates that the typical patient with vascular injury is a man between 20 and 40 year old. In did, man composed 91.66% of all our patients and 54.54% of them were in this age group. Female patients, on the other hand, composed only 8.34% of all patients and contrary to the male they were almost equally distributed between age groups. Such distribution is documented by other authors, as well, and reflects the behavioral characteristics of this particular group [1-5].

*Second,* mechanism of injury was shown of major importance for the outcome. In our study, the mechanism of arterial injury was stabbing in 46.66%, gunshot in 31.66%, blunt in 13.33%, and landmine in 8.33%. Blunt injuries and injuries inflicted by gunshot injuries and land mines were the most fatal ones for our patients. Of four fatalities, three (75%) were in the group that suffered gunshot injury and one in the group that suffered blunt injury (25%). On the other hand of seven primary amputations, six (85.72%) were in the group of patients injured by landmines and one (14.28%) in the group with gunshot injury.

Mechanism of injury varies between different countries and, certainly when comparing the situation in peace and war. As found by Magee et al. [6], with the exception of Northern Ireland, vascular trauma is not only uncommon in the U.K, but it differs by the mechanisms of injury from the U.S.A. There are an estimated 200 million guns in the U.S.A. of which 60 million are hand guns and 3 million are assault rifles. Firearms are present in 50% of American households [7]. Firearms are still rare in British homes. A typical review of vascular trauma in a major U.S.A. city, Boston, reported that gunshot wounds accounted for 50% and stabbings for 25% of vascular injuries [8]. There were no gunshot wounds in Oxford, but 23% of injuries were due to knife wounds [6].

According to United Nations Development Program office in Kosovo (UNDP Kosovo) [9] in year 2006 there were around 400.000 illegal weapons in Kosovo and according to the official statistics 50 thousand hunting guns and almost 15 thousand small guns are officially registered in the country [10]. Since Kosovo has somewhat less than two million inhabitants, this means that almost every fourth Kosovo is armed.

War zones yield many vascular injuries as was seen in Vietnam [11], Bosnia, Croatia [12-14], Serbia [15], Izrael and recent battlefields in Afghanistan and Iraq [16-18].

*Third,* anatomic localization of injury has also shown to be of importance for the outcome. Injuries to the thoracic and abdominal aorta as well of the pulmonary artery were fatal in almost all cases. Except in two injuries to the abdominal aorta, that were successfully managed, all patients, actually, died in theater. This course of the injuries reflects the fact that these vessels are not only to large and therefore the exsanguinations is immediate, but also noncompressible and therefore difficult to treat in preoperative phase.

This is the reason why the best injuries to treat are shown to be those of the upper limbs and lower limbs. Of these, the worst to treat are injuries to the popliteal artery. This is not only our experience but was thoroughly discussed in the literature.

*Forth,* associated injuries are determinant for the outcome of the injury. In our study, almost every forth injury to the vessel was complex (24.2%) - associated with the injury to the distant organs or injuries to the veins, nerves or bones in the proximity. Such were all blunt and landmine injuries, 34.21% of the gunshot injuries and only 5.35% of the injuries inflicted by sharp objects. Evaluated statistically difference was important (*X*^2^-test = 16.5, P = 0.001).

Because of these injuries, the reconstruction of the injured vessels had to be delayed (until injuries to vital organs were taken care of) or lasted longer (until other injuries are taken care of). In the first case, prolonged ischemia of the tissues led to an undesirable outcome, and in the second, the infection rate was higher and functional outcome poorer.

*Fifth,* decision to operate, based on the presence of “hard signs” of vascular trauma, has been proved safe in our study. In last five years, we used triplex scan routinely in all our patients and we have found this diagnostic tool very important in supporting clinical decision. This diagnostic approach has shown very effective, since only two injuries, later presented as false aneurysm and arteriovenous fistulas, were missed (Figure 3). It is important to mention however that both of the missed injuries were surgically corrected without sequels.

Beside the fact that we employed fasciotomy in twelve cases (10%), half of whose was prophylactic and that we used the intra-arterial shunts in three occasions (2.5%), these did not change the outcome in our patients.

However, these two damage control techniques are reported to be of use and are a part of the treatment protocols all around the world, including our.

This paper does not discuss employed surgical techniques since they were standardized reflecting recent treatment protocols.

## Conclusion

Blunt injuries, hemodynamic shock at the admission, injury to large thoracic and abdominal vessels and injury to the popliteal artery, associated injuries to vital remote organs and to the vein and the nerve in proximity are factors predictive of poor outcome of the vascular injury.

### Ethical approval and Consent

This study is approved by the Ethical Committee of the University Clinical Center of Kosova.

## Competing interests

The authors declare that they have no competing interests.

## Authors’ contributions

LJ, AB and HR are the part of the team that performed surgeries; TA and VIJ reviewed literature and helped with the discussion. All authors are major contribution to the manuscript. 
